# Computed tomography defined femoral artery plaque composition predicts vascular complications during transcatheter aortic valve implantation

**DOI:** 10.1259/bjr.20230296

**Published:** 2023-10-24

**Authors:** Elliott J. Carande, Tarik S Salim, Alexander Chase, Baskar Sekar, Omar Aldalati, Ahmed Hailan, Ayush Khurana, Dave Smith, Daniel Rhys Obaid

**Affiliations:** 1 Cwm Taff Morgannwg University Health Board, Princess of Wales Hospital, Coity Road, Bridgend, United Kingdom; 2 Swansea Bay University Health Board, Morriston Hospital, Swansea, United Kingdom; 3 Swansea University Medical School, Swansea, United Kingdom

## Abstract

**Objective:**

Vascular and bleeding complications after transcatheter aortic valve implantation (TAVI) are common and lead to increased morbidity and mortality. Analysis of plaque at the arterial access site may improve prediction of complications.

**Methods:**

We investigated the association between demographic and procedural risk factors for Valve Academic Research Consortium (VARC-3) vascular complications in patients undergoing transfemoral TAVI with use of a vascular closure device (ProGlide^®^ or MANTA^®^) in this retrospective cohort study. The ability of pre-procedure femoral CT angiography to predict complications was investigated including a novel method of quantifying plaque composition of the common femoral artery using plaque maps created with patient specific X-ray attenuation cut-offs.

**Results:**

23 vascular complications occurred in the 299 patients in the study group (7.7%). There were no demographic risk factors associated with vascular complications and no statistical difference between use of closure device (ProGlide^®^ *vs* MANTA^®^) and vascular complications. Vascular complications after TAVI were associated with sheath size (OR 1.36, 95% CI 1.08–1.76, *P* 0.01) and strongly associated with CT-derived necrotic core volume in the common femoral artery of the procedural side (OR 17.49, 95% CI 1.21–226.60, *P* 0.03).

**Conclusion:**

Plaque map analysis of the common femoral artery by CT angiography reveals patients with greater necrotic core are at increased risk of VARC-3 vascular complications.

**Advances in knowledge:**

The novel measurement of necrotic core volume in the common femoral artery on the procedural side by CT analysis was associated with post-TAVI vascular complications, which can be used to highlight increased risk.

## Introduction

Transcatheter aortic valve implantation (TAVI) is an alternative to surgical aortic valve replacement, frequently used in high-, and intermediate-risk patients.^1–5^ Advances in the procedure and technology including vascular closure devices (VCDs), such as the suture based ProGlide^®^ and plug-based MANTA^®^ devices allow it to be performed entirely percutaneously. However, complications associated with vascular access^6–11^ and bleeding^12–18^ still occur and result in significant increases in morbidity and mortality. Therefore, the Valve Academic Research Consortium (VARC-3) have defined clinical end points for vascular access site and access-related complications, bleeding and transfusion.^19,20^

Previous studies have investigated demographic and procedural risk factors associated with VARC Vascular Access Site & Access-Related Complications, demonstrating that female sex^21^ and sheath to femoral artery ratio (SFAR)^6^ and anatomical features such as distance from common femoral artery (CFA) to skin^22^ and tortuosity^23^ are significantly associated with an increased risk of VARC vascular complications. Arterial calcification defined by CT imaging is also a risk factor for vascular complications.^6,22,24^ However, to date only qualitative measures of calcification have been utilised. The role of other non-calcified plaque components as a risk factor for VARC-3 vascular access site and access-related complications is not known.

We have previously described a technique utilising CT angiography lumen contrast/plaque attenuation ratios that can discriminate calcified plaque, fibrous plaque and necrotic core in both the coronary,^25^ and carotid arteries.^26^ In this study, we investigate whether identifying quantitative plaque composition from CT-derived plaque maps of the CFAs created using these ratios predicted risk of VARC-3 Vascular access site and access-related complications in patients undergoing transfemoral TAVI access with VCD closure.

## Methods and materialsS

### Patient selection and procedure

In this single centre, retrospective cohort study all sequential patients who had undergone transcatheter aortic valve implantation (TAVI), between 21 March 2017 and 12 August 2020 (*n* = 333) at our institute were considered for inclusion. For inclusion into the study, patients were required to have undergone both a CT planning scan (including the iliofemoral arteries), and then felt to be suitable percutaneous transfemoral access and closure with a vascular closure device (VCD).

**Figure 1.**
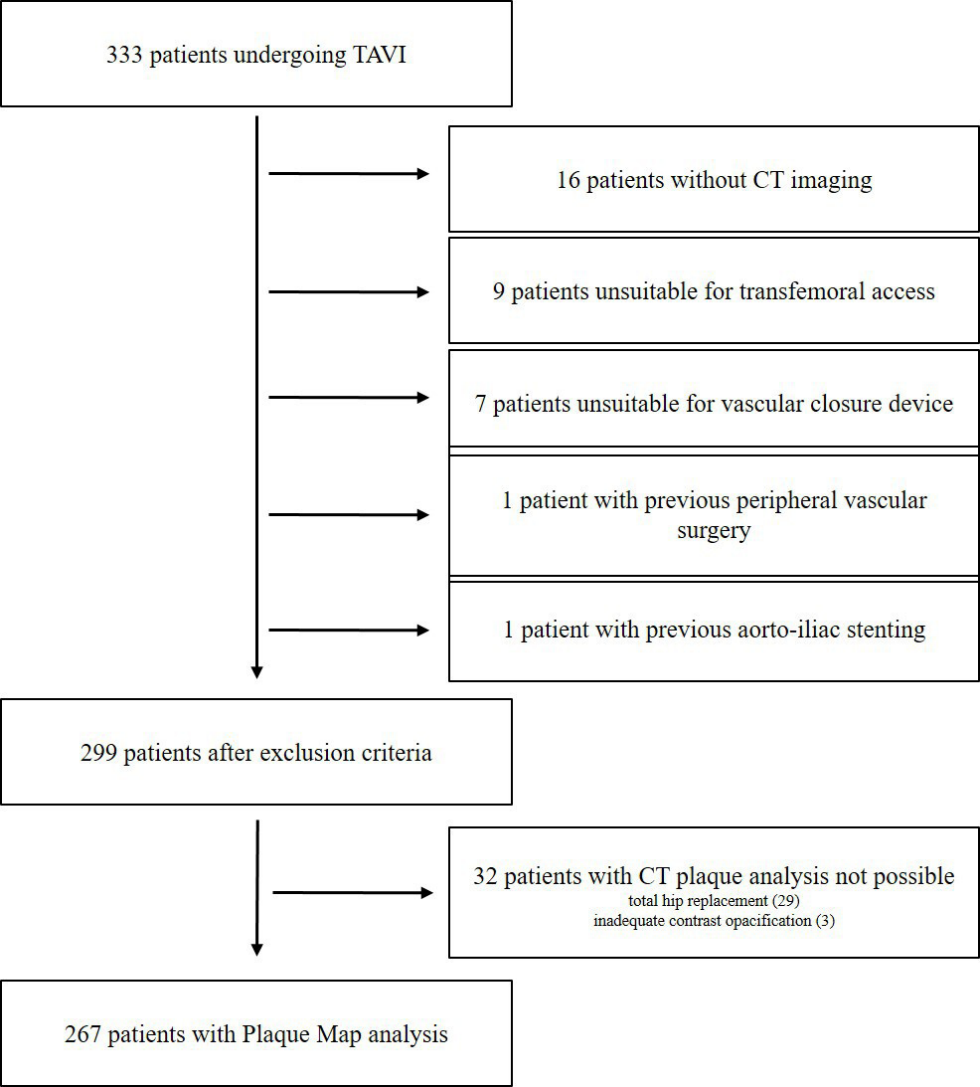
Exclusion criteria. TAVI, transcatheter aortic valve implantation.

16 patients were excluded as their iliofemoral arteries were imaged by catheter angiography, not CT. Patients were considered ineligible for transfemoral access if the ileofemoral diameter was ≤5 mm due to atherosclerotic plaque or small calibre arteries and these underwent alternative access TAVI (*n* = 9) and were excluded. Patients were excluded from VCD if there was anterior wall calcification in the common femoral at the anticipated puncture site of the CFA (*n* = 7). Finally, patients were excluded from the study if they had previous peripheral vascular surgery (*n* = 1) or stenting (*n* = 1). Therefore, 299 patients were included after the above exclusion (Figure 1). The VCD device (ProGlide^®^ or MANTA^®^) was chosen at the operators’ discretion.

The side of large-bore device insertion was determined by the operator after reviewing the CT images to obtain a combination of maxim lumen diameter and minimal arterial disease and tortuosity. Ultrasound guidance was used to identify puncture sites with low plaque burden (both calcified and non-calcified) and the location in the CFA was confirmed fluoroscopically. Patients undergoing closure using a MANTA^®^ VCD received a single 18 French device. Patients undergoing closure using ProGlide^®^ received x2 pre-deployed devices as default but could receive additional ProGlide^®^ devices as required to achieve haemostasis. Baseline characteristics were obtained from a dedicated TAVI database, which was completed with patients’ information at the time of the procedure.

### Common femoral artery CT angiography

Patients had a TAVI planning CT angiogram, which consisted of an electrocardiogram (ECG)-gated cardiac CT and a contrast-enhanced helical scan of the whole aorta to below the femoral artery bifurcation to establish procedural feasibility. Images were analysed using Vitrea^®^ Software v. 7.14.5.13 (Vital Images, Inc.).

The anteroposterior diameter of the CFA, including any plaque, was measured manually at the mid-femoral head, whilst distance to skin from the CFA was measured from the most anterior point of the CFA perpendicular to the skin surface at the mid-femoral head. The length of the arterial vessels was measured from the most proximal point of the common iliac artery to the most distal point of the CFA. Tortuosity was expressed as the tortuosity index, which is the true length of these vessels divided by the straight-line distance.

### Plaque composition CT analysis

**Figure 2.**
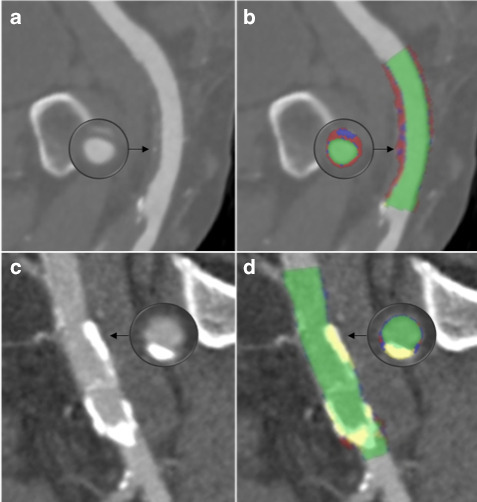
Plaque map analysis of common femoral artery. (a) Common femoral artery with predominantly non-calcified plaque. (b) Plaque map analysis revealing necrotic core (red), fibrous plaque (blue) and lumen (green). (c)Common femoral artery with predominantly calcified plaque. (d) Plaque map analysis revealing calcified plaque (yellow) and lumen (green).

The CFA was analysed on the side of TAVI valve insertion by a single operator (EC). The upper and lower limits were defined with the upper limit the origin of the inferior epigastric artery and the lower limit the most distal point of the CFA prior to the femoral bifurcation. Attenuation (Hounsfield units) of contrast of the lumen was sampled at the mid-femoral head. The attenuation cut-offs for each plaque component were calculated according to ratios of luminal contrast and plaque attenuation (necrotic core <0.21, fibrous plaque 0.21–0.53, calcified plaque >1.54) derived using histological validation and described in detail previously.^26^ This sets attenuation thresholds for plaque components individualised to each patient. These are used by the Vitrea^®^ software to create plaque maps of the CFAs allowing the volumes of the necrotic core, fibrous plaque and calcified plaque to be calculated (Figure 2). To assess the interobserver variability of plaque map analysis, this process was repeated independently by a second operator (DO) in 20 randomly chosen patients.

The artefact created by a hip replacement on the same side as the TAVI procedure interferes with attenuation base plaque analysis, so these patients (29) were excluded from analysis of plaque composition in the study. Some patients (3), despite having sufficient contrast to carry out analysis of CFA dimensions, did not have sufficient contrast for advanced plaque analyse of the CFA. Therefore, 267 patients were included in the analysis of plaque composition.

### End points

#### Vascular access site & Access-Related complications

**Figure 3.**
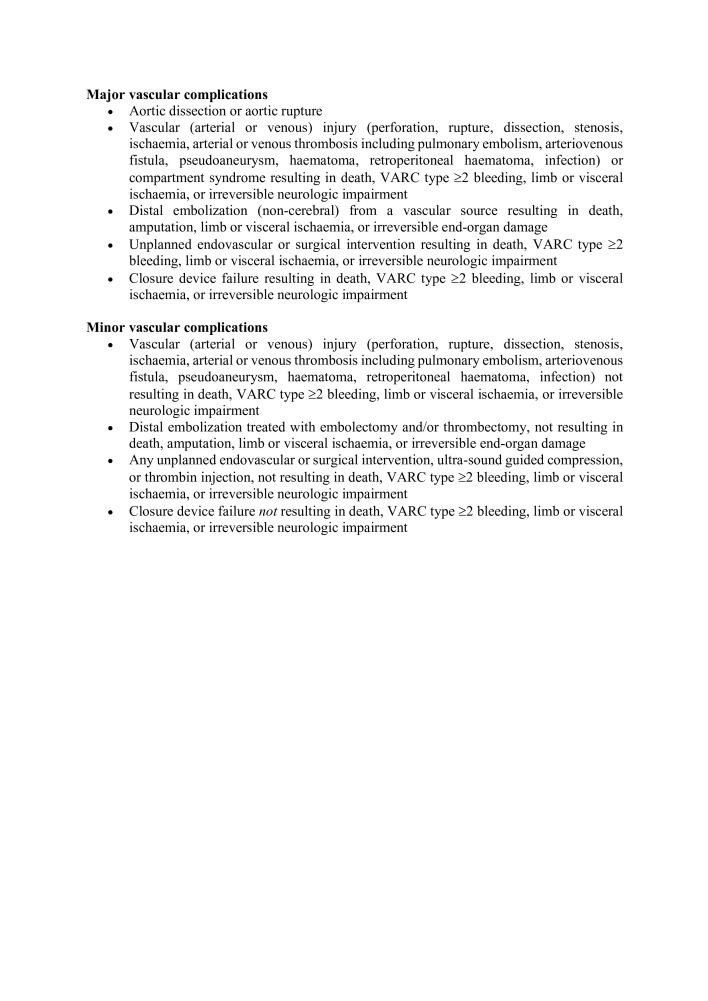
VARC-3 vascular access and access-related complications. Adapted from Généreux et al.^20^

The primary end point of the study was a composite of major vascular complications and minor vascular complications as determined by the VARC-3 vascular access site and access-related complications definitions as shown in Figure 3.

#### Supplemental analysis

Time to haemostasis, which was measured in minutes and defined as the time from end of valve deployment until successful haemostasis obtained at the access site was compared for both VCD used for closure and compared to a historical cohort that underwent the gold standard of surgical closure (Supplementary Material).

Supplementary Material 1.

STROBE Statement.

### Statistical analysis

Statistical analysis was performed using Prism 9, GraphPad (GraphPad Software, San Diego). Unpaired Student’s *t* test was used to compare continuous variables with normal distribution and the Mann–Whitney *U* test was used to compare continuous variables with non-normal distribution. Fisher’s exact test was used to compare proportions of categorical variables. Pearson correlation coefficient was used to compare two continuous variables. Multivariate analysis was carried out using multiple logistic regression for categorical data and multiple linear regression for continuous data. Differences in data were expressed as an odds ratio (OR) with 95% confidence limits (CI). A 2-sided *p* < .05 was deemed significant for all statistical tests.

## Results

### Baseline characteristics

Baseline characteristics for the 299 patients undergoing TAVI procedure with vascular closure devices and CFA dimensions analysis between 1 October 2016 and 12 August 2020 are presented in Table 1. Baseline characteristics for the 267 patients who went on to undergo plaque composition analysis are provided in the Supplementary Material.

**Table 1.** Baseline characteristics

	N	%
**TOTAL**	299	100
Male	145	48.5
Age (years) *Mean ± SD*	83.1 ± 6.9	-
Diabetes	75	25.1
ProGlide^®^	64	21.4
MANTA^®^	235	78.6
Current or Previous Smoker	156	52.2
Baseline Creatinine (μmol/L) *Mean ± SD*	107.8 ± 58.5	-
Previous MI	53	17.7
BMI (kg/m^2^) *Mean ± SD*	26.8 ± 5.6	-
Urgent procedure	95	31.8
Sheath size (Fr) *Mean ± SD*	16.1 ± 2.1	-

SD: standard deviation, MI: myocardial infarction, BMI: body mass index

Of the patients undergoing a TAVI procedure, 235 (78.6%) were closure with the MANTA^®^ device, whilst 64 (21.4%) of patients had closure with ProGlide^®^ devices. The majority of patients receiving ProGlide^®^ devices 53/64 (83%) achieved haemostasis with two devices. Additional ProGlide^®^ devices were required in nine patients (14%), with seven patients requiring three ProGlide^®^ devices and two patients requiring five ProGlide^®^ devices to achieve haemostasis.

145 (48.5%) patients were male and the average age was 83.1 ± 6.9 years (mean ± standard deviation). Overall, there were six in-hospital deaths (2.0%) following the procedural group, of which 2 (0.7%) were related to an access site complication.

There were 11 VARC-3 major events (3.7%) and 12 VARC-3 minor events (4.0%) meaning the primary endpoint was met in 23 patients (7.7%) (Table 2). All events occurred at the primary access site.

**Table 2.** Description of VARC-3 major and minor events, demonstrating vascular closure device used, valve type implanted, valve size implanted and sheath size used in each instance

VARC-3 major events				
**Closure device**	**Valve type**	**Valve size (mm**)	**Sheath size (Fr**)	**VARC-3 major event**
ProGlide^®^	Portico	29	19	Retroperitoneal haematoma resulting in VARC Type 2 bleeding (BARC 3a)
ProGlide^®^	Portico	29	19	Closure device failure resulting in VARC Type 2 bleeding (BARC 3a)
ProGlide^®^	Portico	27	19	Haematoma resulting in VARC Type 3 bleeding (BARC 3b)
ProGlide^®^	Portico	29	19	Pseudoaneurysm with unplanned surgical repair resulting in VARC Type 3 bleeding (BARC 3b)
MANTA^®^	Sapien 3	26	14	Closure device failure resulting in VARC Type 3 bleeding (BARC 3b)
MANTA^®^	Portico	27	19	i.Closure device failure resulting in VARC Type 2 bleeding (BARC 3a) ii.Dissection resulting in VARC Type 2 bleeding (BARC 3a)
MANTA^®^	Sapien 3	23	14	Retroperitoneal haematoma resulting in VARC Type 4 bleeding (BARC 5b)
MANTA^®^	Sapien 3	26	19	Pseudoaneurysm resulting in VARC Type 2 bleeding (BARC 3a)
MANTA^®^	Portico	27	19	Retroperitoneal haematoma resulting in VARC Type 2 bleeding (BARC 3a)
MANTA^®^	Portico	29	19	Closure device failure resulting in VARC Type 4 bleeding (BARC 5b)
MANTA^®^	Sapien 3	26	14	Pseudoaneurysm with unplanned surgical intervention resulting in VARC Type 3 bleeding (BARC 3b)
**VARC-3 minor events**				
**Closure device**	**Valve type**	**Valve size (mm**)	**Sheath size (Fr**)	**VARC-3 minor event**
ProGlide^®^	Portico	29	19	Femoral dissection managed conservatively
ProGlide^®^	Portico	25	18	Femoral thrombus requiring endovascular ballooning
ProGlide^®^	Sapien 3	29	16	Femoral dissection requiring endovascular ballooning
ProGlide^®^	Sapien 3	23	18	Retroperitoneal haematoma resulting in VARC Type 1 bleeding (BARC 2)
MANTA^®^	Sapien 3	23	14	Arterial thrombus managed conservatively, later readmitted with ischaemic limb requiring vascular surgery
MANTA^®^	Portico	29	19	Pseudoaneurysm managed conservatively
MANTA^®^	Portico	25	18	Arterial thrombus managed conservatively
MANTA^®^	Sapien 3	29	16	Femoral dissection requiring endovascular ballooning
MANTA^®^	Sapien 3	23	14	Femoral dissection requiring endovascular ballooning
MANTA^®^	Allegra	27	18	Femoral dissection requiring endovascular ballooning
MANTA^®^	Portico	27	15	Femoral dissection requiring endovascular ballooning
MANTA^®^	Portico	25	14	Femoral dissection & occlusion requiring endovascular ballooning

BARC, Bleeding Academic Research Consortium ; VARC-3, Valve Academic Research Consortium 3.

The VARC-3 major events that occurred were percutaneous closure device failure with fatal bleeding (1), percutaneous closure device failure with life-threatening bleeding (2), percutaneous closure device failure with major bleeding (2), retroperitoneal haematoma with fatal bleeding (1). retroperitoneal haematoma with major bleeding (2), pseudoaneurysm with major bleeding (1), pseudoaneurysm requiring surgical repair and major bleeding (1), and pseudoaneurysm requiring surgical repair (1).

The VARC-3 minor events that occurred were femoral dissection requiring endovascular ballooning (5), femoral occlusion requiring endovascular ballooning (1), femoral dissection and occlusion requiring endovascular ballooning (1), femoral dissection managed conservatively (1), retroperitoneal haematoma with minor bleeding (1), arterial clot managed conservatively (2), and small pseudoaneurysm of femoral artery managed conservatively 1).

### Demographic and procedural risk factors for composite outcome

Comparison of demographic risk factors did not demonstrate any significant associations with increased number of VARC-3 outcomes (Table 3). Regarding procedural risk factors, we found that there was no significant association with the use of ProGlide^®^ over MANTA^®^, the urgency of TAVI procedure, the side of transfemoral access, or hip replacement having been previously performed on the same side as TAVI access (Table 3). We found a significant difference with an increased sheath size used in the procedure and the composite of VARC-3 outcomes (T 2.29, p 0.02).

**Table 3.** Demographic risk factors for VARC-3 composite outcome

	No VARC-3 outcome	%	VARC-3 composite outcome	%	Univariate analysis	95% CI	*p-* value
**TOTAL**	276	–	23	–	–	–	–
Male	136	49.3%	9	39.1%	OR 0.66	0.26–1.57	P 0.39
Age (years) *Mean ± SD*	83.1 ± 7.0	–	84.3 ± 5.4	–	T 0.85	–	P 0.40
Diabetes	70	25.4%	5	21.7%	OR 0.82	0.32–2.24	P 0.81
Current/Previous smoker	143	51.4%	13	56.5%	OR 1.21	0.51–2.82	P 0.83
Baseline creatinine (μmol/L) *Mean ± SD*	108.1 ± 60.4	–	105.3 ± 25.0	–	T 0.22	–	P 0.83
Previous MI	46	16.7%	7	30.4%	OR 2.19	0.85–5.61	P 0.15
BMI (kg/m^2^) *Mean ± SD*	26.7 ± 5.6	–	27.8 ± 5.6	–	T 0.90	–	P 0.37
ProGlide^®^	56	20.3%	8	34.8%	OR 2.10	0.87–5.16	P 0.11
Urgent procedure	87	31.5%	8	34.8%	OR 1.16	0.49–2.79	P 0.82
Right transfemoral access	207	75.0	18	78.3%	OR 1.20	0.44–3.05	*p* > 0.99
Procedural side-hip replacement	25	9.1%	4	17.4%	OR 2.11	0.73–6.38	P 0.26
Sheath size (Fr) *Mean ± SD*	16.1 ± 2.0	–	17.1 ± 2.2	–	T 2.29	–	P 0.02*

BMI, body mass index ; MI, myocardial infarction; SD, standard deviation; VARC-3, Vascular Access-Related Complication.

### Common femoral artery dimensions

Similarly, regarding our analysis of CFA dimensions on CT images, there was no significant association found between the composite of VARC-3 outcomes and diameter of the CFA, perpendicular distance from CFA to skin, or tortuosity of the vessel (Table 4).

**Table 4.** Analysis of CFA dimensions as a risk factor for composite outcome

	No VARC-3 outcome	VARC-3 composite outcome	Univariate analysis	*p-*value
**TOTAL**	276	23	–	–
CFA diameter (mm) *Mean ± SD*	8.4 ± 1.6	7.9 ± 1.4	T 1.44	P 0.15
Distance to skin (mm) *Mean ± SD*	40.9 ± 21.4	46.3 ± 20.4	T 1.16	P 0.25
Tortuosity *Mean* ± *SD*	1.2 ± 0.1	1.2 ± 0.1	T 0.25	P 0.80
SFAR *Mean* ± *SD*	0.65 ± 0.16	0.74 ± 0.17	T −2.49	P 0.013

CFA: common femoral artery, SD: standard deviation, SFAR: sheath size to femoral artery ratio;VARC-3, Vascular Access-Related Complication.

### Plaque composition and composite outcome

By identifying the composition of plaque in the vessel, we found a significant association between the volume of necrotic core identified and the composite of VARC-3 outcomes in the common femoral artery (T 2.02, *p* 0.04). We did not find any significant association between volume of vessel, plaque burden, total plaque volume, volume of fibrous plaque, or volume of calcification with the composite of VARC-3 outcomes (Table 5). To prevent multicollinearity, only one of sheath size or SFAR could be entered into the multivariate analysis. Given that CFA diameter was not associated with vascular complications in univariate analysis we elected to use sheath size. After multivariate analysis, we found that sheath size (OR 1.36, 95% CI 1.08–1.76, *p* 0.01) was associated with an increased risk of vascular composite outcome but the strongest predictor was volume of necrotic core in the common femoral artery (OR 17.49, 95% CI 1.21–226.60, *p* 0.03) (Table 6). The repeat analysis of plaque map composition by a second operator to determine interobserver variability revealed necrotic core volume had a bias of −0.032 cm^3^ with 95% limits of agreement from −0.122 to + 0.058 cm^3^. Fibrous plaque volume bias was 0.002 cm^3^ with 95% limits of agreement from −0.137 to + 0.142 cm^3^. Calcified plaque volume bias was 0.012 cm^3^ with 95% limits of agreement from −0.091 to + 0.114 cm^3^. Bland–Altman plots are provided in Supplementary Material.

**Table 5.** Plaque composition of common femoral artery as risk factor for VARC-3 composite outcome

	No VARC-3 outcome	VARC-3 composite outcome	Univariate analysis	*p-*value
**TOTAL**	248	19	-	-
Volume (cm^3^) *Mean ± SD*	4.14 ± 1.67	3.79 ± 2.21	T 0.86	P 0.39
Plaque burden (%) *Mean ± SD*	24.47 ± 10.49	23.17 ± 5.48	T 0.53	P 0.60
Total plaque volume (cm^3^) *Mean ± SD*	1.00 ± 0.62	0.85 ± 0.34	T 1.09	P 0.28
Necrotic core volume (cm^3^) *Mean ± SD*	0.17 ± 0.13	0.24 ± 0.25	T 2.02	P 0.04*
Fibrous plaque volume (cm^3^) *Mean ± SD*	0.42 ± 0.18	0.37 ± 0.15	T 1.20	P 0.23
Calcification volume (cm^3^) *Mean ± SD*	0.42 ± 0.58	0.23 ± 0.21	T 1.41	P 0.16

SD: standard deviation;VARC, Vascular Access-Related Complication.

**Table 6.** Multivariate analysis of risk factors for VARC-3 composite outcome

	No VARC-3 outcome	VARC-3 composite outcome	Multivariate analysis	95% CI	*p-*value
**TOTAL**	248	19			
Sheath size (Fr) *Mean ± SD*	16.05 ± 2.05	17.21 ± 2.07	OR 1.36	1.08–1.76	P 0.01*
Necrotic core volume (cm^3^) *Mean ± SD*	0.17 ± 0.13	0.24 ± 0.25	OR 17.49	1.21–226.60	P 0.03*

SD: standard deviation;VARC-3, Vascular Access-Related Complication.

## Discussion

In our study of demographic factors and femoral artery plaque composition as a risk factor for vascular complications, we found a significant association between increased size of outer sheath diameter, as well as the necrotic core volume in the CFA, with vascular complications during TAVI procedures. Our finding of increased sheath size as a risk factor is of interest for other vascular procedures using large sheaths, such as EndoVascular Aneurysm Repair, particularly given the reported complication rate of this procedure between 16 and 30%^27^ and it is possible that quantifying CFA necrotic core may also be of use in these procedures.

There were no significant associations between demographic risk factors and vascular complications in our study. We note that a previous study^12^ has identified female sex as a risk factor of vascular complications after TAVI. We did find that increased sheath size was a risk factor for vascular complications at both uni- and multivariate level of analysis.

Larger sheath size has previously been reported as a risk factor for vascular complications,^12^ whilst other studies have also demonstrated a significantly increased risk of vascular complications with an increased SFAR.^6^

Analysis of dimensions with CT imaging, such as CFA diameter, and perpendicular distance from CFA to skin was not a significant risk factor for the composite of VARC-3 major and minor vascular complications. Importantly, we found that an increase in the total necrotic core volume throughout the CFA was an independent risk factor for vascular complications after TAVI.

Although qualitative femoral calcification has previously been reported as a risk factor for vascular complications,^6,22,24^ this study investigated a novel *quantitative* method of measuring femoral plaque. Despite finding an association with necrotic core volume and VARC-3 outcomes, we did not find a significant association between the volume of calcification and VARC-3 composite outcome. A possible explanation for this finding may be that calcified plaque is a risk factor only if it is present on the anterior wall of the CFA and percutaneous closure was avoided in these patients. Given that anterior calcified plaque precluded selection for percutaneous closure but anterior non-calcified plaque did not this may have influenced the results. Another possibility is that the presence of calcified plaque acts as a surrogate for other plaque types, a feature seen in the coronaries.^28^

The use of plaque map improves the classification of plaque composition and our study demonstrates a strong association between necrotic core and VARC-3 outcomes (OR 17.49), this suggests that CFA plaque necrotic core may be a key predisposing factor to vascular complications. Previous studies have revealed that coronary plaques with high necrotic core content “thin-cap fibroatheroma” are the most likely to rupture^29,30^ and their equivalent in the CFA may have a predisposition to disruption during the stress of large bore access and instrumentation. It has been demonstrated that the components of necrotic core (lipid and thrombus) are less stiff with lower resistance to deformation than fibrous tissue and arterial media.^31^ For plaque map analysis of necrotic core to be utilised clinically, it should be generalisable to other centres that may have other reporting software, have acceptable interobserver variability and not be too time-consuming. This study was performed with Vitrea^®^ software, however, we have previously demonstrated similar plaque maps in the coronary arteries using a different software vendor.^25^ Once the attenuation of luminal contrast is known the ratios described can be utilised to calculate plaque volume on any software where the attenuation thresholds for quantifying plaque can be altered. Our interobserver analysis revealed acceptably low variability with narrow levels of agreement. This mirrors results in coronaries where plaque map analysis identified high-risk plaque with lower interobserver variability than conventional CT coronary angiography features.^32^ Performing plaque map analysis using the Vitrea^®^ software requires around 5 min per patient.

In this study, we found that there was a vascular complication (as per the VARC-3 criteria^20^) in 7.7% of patients. This was lower than the 21% recorded in two other studies,^22,33^ This may be due in part to the mandated use of ultrasound-guided puncture and the exclusion of VCD use in patients with common femoral artery anterior wall calcification. The complication rate was similar (6.6%) to a recent study comparing vascular closure with MANTA^®^ or ProGlide^®^.^34^

In contrast to the above study, which found a significantly lower rate of vascular complications in the ProGlide^®^ group compared to the MANTA^®^ group, we found no significant difference in the percentage of vascular complications using either device (ProGlide^®^ (9.7%) or MANTA^®^ (5.7%), *p* = 0.11). Our results are supported by other data, which has demonstrated no significant difference between closure devices and vascular complications.^35^ Conversely, the recent CHOICE-CLOSURE study demonstrated an increased risk of VARC-2 vascular complications in the MANTA^®^ group compared to the ProGlide^®^ group.^36^

We noted that similar types of complications were encountered with the use of each device, with percutaneous closure device failure, retroperitoneal haematoma and pseudoaneurysm occurring in both device types. Similarly, with regards to VARC-3 minor events, femoral dissections and femoral occlusions occurred in both device groups.

### Limitations

There were some limitations to this study. Firstly, diagnosis of plaque composition in this study was only radiological, and was not supported by other means, such as intravascular imaging. However, our method has been utilised in previous work, which has demonstrated the suitability of CT imaging with attenuation ratios to classify different compositions of coronary plaque validated against the gold-standard of post-mortem histology.^25^

Secondly, the sheaths used for deployment of the Sapien three valve (Edwards Lifesciences) incorporate a dynamic expandable technology, so the final actual outer diameter will be larger than that stated following valve deployment.^37^ Thirdly, we defined arterial size as the anteroposterior diameter of the CFA at the level of the mid-femoral head. This was chosen as we felt it would obtain a standardised, repeatable measurement. However, it is possible that different diameters may have been obtained from cross-sectional analysis. Fourthly, we found a high variability in the CT data with wide CIs. However, these statistically significant findings remained after multivariate analysis had been performed. Finally, patients with VCD closure were recruited for this study sequentially and therefore were not randomised. Therefore, despite the use of multivariate analysis, bias cannot be excluded in this study.

## Conclusion

CT defined femoral artery plaque composition utilising plaque maps predicted vascular complications during transcatheter aortic valve implantation. Necrotic core, rather than calcified plaque volume was associated with a significant increase in VARC vascular complications along with increased sheath size.
