# Supplementary material for: Computed tomography defined femoral artery plaque composition predicts vascular complications during transcatheter aortic valve implantation
Source: Br J Radiol. 2023 Oct 24;96(1152):20230296. doi: 10.1259/bjr.20230296 (PMC10646661; doi:10.1259/bjr.20230296)

**SUPPLEMENTARY MATERIAL**

**Comparison of Vascular Closure Devices & Surgical Closure**

The patients undergoing percutaneous transfemoral, percutaneous aortic valve implantation (TAVI) with vascular closure device (VCD) were compared to a historical cohort of patients that underwent the previous gold standard of surgical arteriotomy closure. The demographics are listed in **Supplemental Table 1**.

**Supplemental Table 1:** Baseline characteristics for surgical cohort group

|  | **N** | **%** |
| --- | --- | --- |
| **TOTAL** | 60 | 100 |
| Male | 26 | 43.3 |
| Age (years)  *Mean* ± *SD* | 82.7 ± 7.9 | - |
| Diabetes | 9 | 15.0 |
| Current or Previous Smoker | 34 | 56.7 |
| Baseline Creatinine (μmol/L)  *Mean* ± *SD* | 136 ± 124.5 | - |
| Previous MI | 20 | 33.3 |
| BMI (kg/m^2^)  *Mean* ± *SD* | 26.9 ± 5.4 | - |
| Urgent Procedure | 24 | 40.0 |
| Sheath Size (Fr)  *Mean* ± *SD* | 14.8 ± 1.5 | - |

SD: standard deviation, MI: myocardial infarction, BMI: body mass index

*Time to haemostasis*

The secondary outcome from the study was the time to haemostasis, which was measured in minutes and defined as the time from valve deployment until adequate haemostasis was confirmed by digital subtraction angiography. Patients in whom VCD was used for closure were also compared to the historical cohort that underwent the gold standard of surgical closure to compare vascular complication rates and time to haemostasis.

*Supplemental Results*

***Vascular Composite Outcomes Between Vascular Closure Devices & Surgical Closure***

There was no significant difference between the frequency of composite outcomes occurring in the vascular closure device (7.3%) group compared to surgical closure (5.0%) group (OR 1.49, 95% CI 0.49 – 4.85, P 0.78).

***Time to Haemostasis Between Vascular Closure Devices***

ProGlide^®^ closure patients had a longer time to haemostasis (21.1 ± 14.8 minutes) compared to MANTA^®^ (16.8 ± 8.8 minutes) closure (Z -2.80, p 0.005). After multivariate analysis of all demographic risk factors, procedural risk factors, as well as preliminary and plaque composition computed tomography (CT) analysis, we found a significant association only between the use of ProGlide^®^ closure and increased time to haemostasis (T 2.99, p 0.04).

***Time to Haemostasis Between Vascular Closure Devices & Surgical Closure***

There was no significant difference between time to haemostasis in ProGlide^®^ closure patients compared with surgical closure (Z 1.74, P 0.08). There was no significant difference between time to haemostasis in the surgical closure and MANTA^®^ closure groups (Z -1.32, P 0.19).

Increased time to haemostasis was only associated with ProGlide^®^ device closure in our study. Time taken for haemostasis was likely shorter for MANTA^®^ than ProGlide^®^ due to intrinsic differences in the methods of deployment and the need for additional ProGlide^®^ deployment in nine cases. Whilst the actual difference is time (4.3 minutes) is unlikely to be clinically relevant, the requirement for additional devices may have cost implications.

**Table S2:** Baseline characteristics of patients that underwent plaque composition analysis

|  | **N** | **%** |
| --- | --- | --- |
| **TOTAL** | 267 | 100 |
| Male | 125 | 46.8 |
| Age (years)  *Mean* ± *SD* | 83.2 ± 7.0 | - |
| Diabetes | 71 | 26.6 |
| ProGlide^®^ | 61 | 22.8 |
| MANTA^®^ | 206 | 77.2 |
| Current or Previous Smoker | 139 | 52.1 |
| Baseline Creatinine (μmol/L)  *Mean* ± *SD* | 108.7 ± 60.7 | - |
| Previous MI | 48 | 18.0 |
| BMI (kg/m^2^)  *Mean* ± *SD* | 26.7 ± 5.6 | - |
| Urgent Procedure | 88 | 33.0 |
| Sheath Size (Fr)  *Mean* ± *SD* | 16.1 ± 2.1 | - |

SD: standard deviation, MI: myocardial infarction, BMI: body mass index

**Supplementary Figure 1:** Bland-Altman analysis of interobserver variability of plaque map analysis


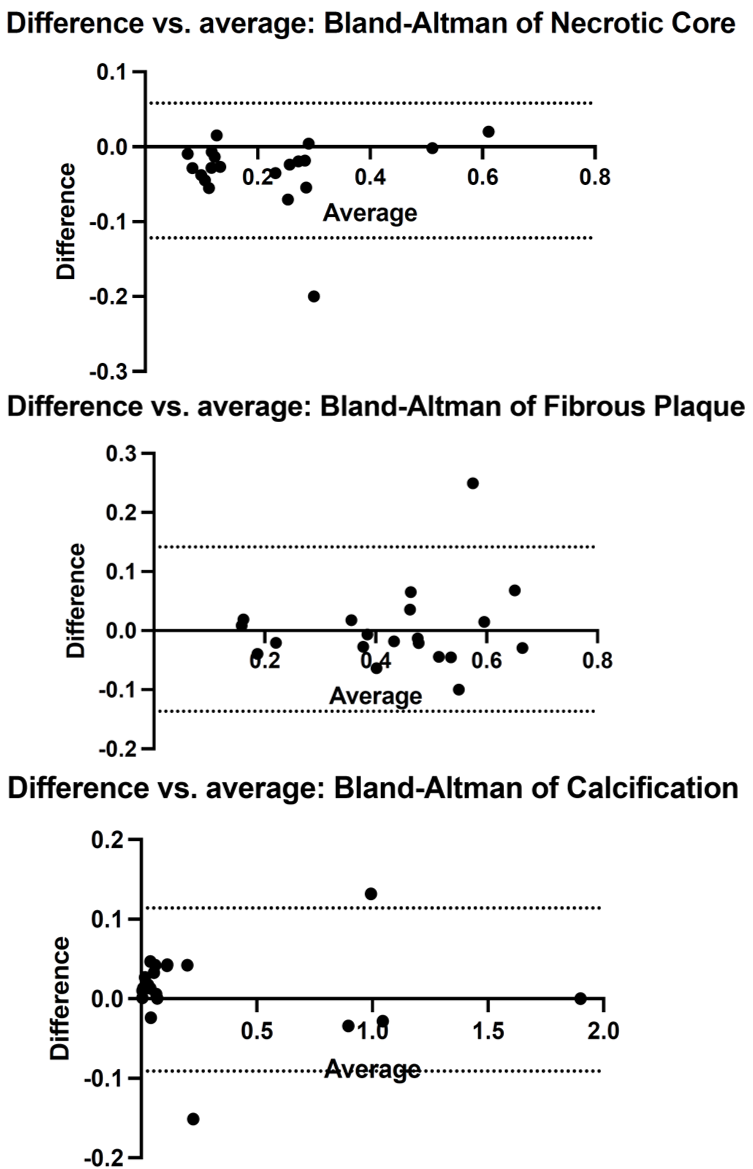

Supplement: Supplementary Material 1. [file bjr.20230296.suppl-01.docx]
